# Computational Evaluation of the Fracture Behavior of Porous Asphalt Concrete Exposed to Moisture and Salt Erosion

**DOI:** 10.3390/ma17071505

**Published:** 2024-03-26

**Authors:** Yuheng Liang, Jiaqi Chen, Liang Li

**Affiliations:** Department of Civil Engineering, Central South University, Changsha 410075, China; yuheng.liang@csu.edu.cn (Y.L.); chenjiaqi@csu.edu.cn (J.C.)

**Keywords:** salt erosion, porous asphalt concrete (PAC), fracture behavior, cohesive zone model, finite element model

## Abstract

Salt erosion has an adverse impact on the durability of asphalt pavements. Porous asphalt concrete is particularly susceptible to the influence of salt. In this study, a finite element model was developed to investigate the fracture behavior of PAC exposed to salt erosion. The 2D heterogeneous structure of PAC was generated with an image-aided approach to computationally study the fracture behavior of PAC. Laboratory SCB tests were conducted to validate the finite element model. The simulation results of the SCB tests indicate that the peak load of the PAC decreased by 21.8% in dry-wet cycles and 26.1% in freeze-thaw cycles compared to the control group. The salt solution accelerated the degradation of the durability of PAC under both dry-wet cycles and freeze-thaw cycle conditions, which is consistent with laboratory tests. After flushing treatment before the drying phase, the peak load of the PAC in salt environments increased by 5.3% compared to that of the samples with no flushing. Salt erosion also results in a higher average value of scalar stiffness degradation (SDEG), and the damaged elements were primarily the cohesive elements in the fracture of the PAC. Additionally, the influence of crucial factors including the void content, adhesion and cohesion, and loading rate on the fracture behavior of the PAC was analyzed. As the void content increases, the average SDEG value of the cohesive elements increases and surpasses the average SDEG value of the adhesive elements at a void content of approximately 9%. The performance of the fine aggregate matrix (FAM) has a much greater impact than the FAM-aggregate interface on the durability of the PAC. And there were more damaged CZM elements with the increase in the loading rate. Salt erosion results in higher SDEG values and a larger number of cohesive damaged elements at each loading rate.

## 1. Introduction

The infiltration of moisture and salt is closely related to the deterioration of urban infrastructure [1,2,3,4], especially for asphalt pavements exposed to de-icing agents in winter [5,6,7,8]. Salt solution penetrating the asphalt pavement could generate moisture damage and erosion, resulting in ravelling, spalling, cracking, asphalt hardening, and other types of damage [9]. Previous studies indicate that, compared to dense-graded asphalt concrete, porous asphalt concrete (PAC) is more susceptible to the effects of salt [10]. Therefore, understanding the deterioration mechanism of PAC under salt erosion is important for durable PAC pavement design.

Experimental methods provide a straightforward way to investigate the impact of salt erosion on the structure and mechanical properties of asphalt binder and asphalt mixture. The mechanisms of the damage caused by salt erosion on asphalt were revealed at the molecular scale utilizing molecular dynamics (MD) [11]. Scanning electron microscopy (SEM) tests, energy-dispersive spectrometry (EDS) tests, and Fourier transform infrared spectroscopy (FTIR) tests are commonly used to study the change in material components and micro-structure in the salt dissolution environment [12,13]. Moreover, the dynamic shear rheometer (DSR) test and surface free energy (SFE) test have been used to study the changes in the properties of asphalt after its treatment with salt solution [14,15]. The results indicate that the salt migrates into the asphalt samples, resulting in the ageing of asphalt and a decrease in the cohesion work of the asphalt. As an important part of asphalt concrete, the performance change in the asphalt will also affect the performance of the asphalt concrete. Cantabro loss tests [12], rutting tests [13], and bending tests [16] have been used to study the deterioration of the mechanical properties of asphalt concrete in a salt erosion environment. In order to analyze the effect of salt erosion on both the asphalt and its mixture, Chen et al. [17] combined the asphalt binder scale, asphalt–aggregate scale, and asphalt concrete scale to analyze the moisture damage resistance of asphalt pavement in salt and acid erosion environments. Previous studies have been able to effectively analyze the damage caused by salt erosion on dense-graded asphalt concrete. However, PAC is more susceptible to the effects of salt erosion compared to dense-graded asphalt concrete [10]. Therefore, more attention needs to be paid to the influence of salt erosion on the durability of PAC

Due to the high void content, PAC is more susceptible to external factors such as salt, moisture, and temperature, resulting in reduced durability [18,19,20]. The crack resistance of PAC is an important indicator of its durability [21]. Semi-circular bending (SCB) tests have been employed to evaluate the fracture behavior of PAC [22]. To further investigate the internal fracture behavior of asphalt concrete, pull-off tests evaluating the fracture performance at the asphalt–aggregate interface have been widely utilized [23,24,25,26,27]. This method has also been employed to investigate the damage to the asphalt–aggregate interface under the influence of salt and freeze–thaw cycles [28]. Within the asphalt concrete, the performance of asphalt mortar also needs attention. Wang et al. [29], focusing on the internal structure of PAC, utilized improved bonded bitumen strength (BBS) tests to evaluate the influence of asphalt properties, temperature, and moisture on the adhesion/cohesion of asphalt, mortar, and mastic. These experiments provide macro indicators for evaluating the fracture behavior of PAC, but it is difficult to use these experiments to study the damage to its internal structure. Numerical simulations can perform convenient calculations under different conditions and can provide some meso-damage data that cannot be obtained by laboratory tests. It is important to combine experiments and numerical simulations to explore the effect of salt on PAC. 

With the development of computer technology, numerical simulations have been used to study the fracture behavior of asphalt concrete [30,31]. However, the void contents of PAC are usually higher than 18% [32], which may lead to difficulties in model development. X-ray CT scanning reconstruction technology has been widely utilized to develop a model of PAC for mechanical analysis [33]. A method of developing 2D random PAC structures using the discrete element model has been proposed [34]. This method can replicate the intricate internal structure of PAC. However, the impact of salt erosion has not been taken into consideration in previous numerical simulations of PAC. Therefore, a 2D heterogeneous model of PAC was developed, which provides a computational evaluation of the influence of salt erosion on the fracture behavior of PAC. 

## 2. Objectives

The objective of this study is to investigate the effect of salt erosion on the fracture behavior of porous asphalt concrete based on meso-scale computational modeling and finite element analysis. The developed model used an image-assisted approach to generate the heterogeneous model structures of the porous asphalt concrete and utilized the cohesive zone model for the fracture analysis. Pull-off tests and SCB tests were conducted to determine the necessary material parameters under different salt erosion environments. The fracture process of the porous asphalt concrete was simulated with the developed finite element model and was validated with the experiment. Utilizing the validated model, the effect of salt erosion on the fracture behavior of porous asphalt concrete was computationally investigated.

## 3. Materials and Experiments

The pull-off tests were conducted to obtain the bond strength of the PAC. The SCB tests were conducted to obtain the fracture energy of the fine aggregate matrix (FAM) and peak load of PAC, respectively. To simulate the salt erosion environment, the specimens were subjected to freeze–thaw cycles and wet–dry cycles using a salt solution. 

### 3.1. Materials

In this study, PAC was treated as a composite material consisting of FAM, coarse aggregates (particle size > 1.18), and air voids. The FAM was a mixture of asphalt binder, mineral fillers, and fine aggregates (particle size ≤ 1.18). The gradation of PAC and its FAM are presented in Table 1. The target void content of the PAC was 20%, and the optimum binder content (OBC) was 6.5%. The coarse and fine aggregates used in the experiments were both basalts. The physical properties of asphalt and basalt are presented in Table 2 and Table 3.

### 3.2. Test Methods

#### 3.2.1. Pull-Off Tests

**Figure 1:**
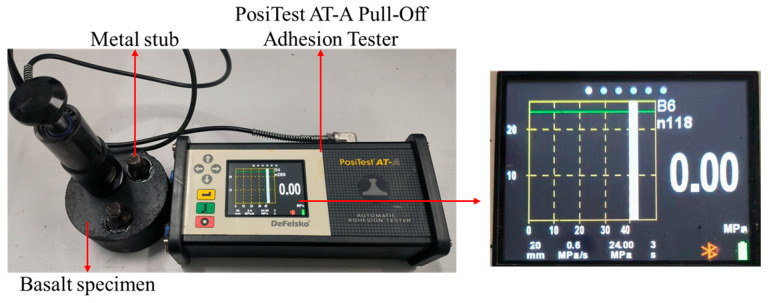
Pull-off tests.

Adhesive strength (bonding strength of the interface between FAM and aggregates) and cohesive strength (bonding strength of the interface between FAM and FAM) can be obtained by pull-off tests. The pull-off test [36] system used in this study includes the PosiTests AT-A Pull-Off Adhesion Tester, basalt specimens, and metal pull-off stubs (Figure 1). This instrument can generate a constant rate of pulling pressure to separate the metal stub from the basalt specimen to measure the pull-off tensile strength (POTS). By observing the fracture surface of PAC, two types of failure modes were identified: adhesive failure at the asphalt–aggregate interface, and cohesive failure within the asphalt. The adhesive and cohesive strength obtained by pull-off tests and post-processing of test data will be described in this section. 

The experimental steps for the pull-off tests were as follows [36]:

(1) Preparation. Placed asphalt, metal stubs, and basalt specimens in an oven at 170 °C to ensure that the temperature is consistent when making the test samples. The metal stub has a diameter of 20 mm, and the basalt specimen has a height of 50 mm and a diameter of 100 mm.
(2) Making. The heated metal stubs were uniformly coated with the substrate asphalt at its bottom and then placed on the surface of the basalt specimens. Moreover, the prepared samples were conditioned at 25 °C for 24 h until the asphalt fully solidified.
(3) Testing. The samples were placed in an environmental chamber at −10 °C for 4 h. The loading rate of the pull-off tests was 0.6 MPa/s.

**Figure 2:**
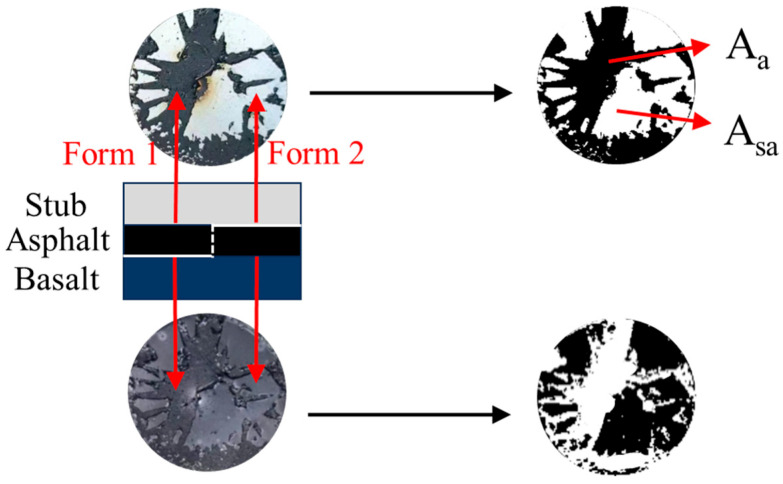
Specimen image and binary image.

The pull-off tests and the associated fracture interface are shown schematically using a digital camera. There were two forms between the stubs and basalt specimens as shown in Figure 2. The first form was adhesive failure between asphalt and basalt when there was asphalt on the stub and no asphalt on the basalt. The second form was adhesive failure between asphalt and stub when there was no asphalt on the stub and asphalt on the basalt. 

The photos taken by the camera were binarized as shown in Figure 2 and the proportion of each failure form could be determined by the binary image. However, the asphalt phase is contained by FAM in asphalt mixtures. The adhesive strength of the FAM-aggregate interface is different from the adhesive strength of the asphalt–aggregate interface. The following formulas are proposed to determine the adhesive strength of the FAM-aggregate interface in this study:

(1) $$POTS=\sigma_{a-b}\times A_{a-b}+\sigma_{a-s}\times A_{a-s}$$

(2) $$\sigma_{\mathrm{adh}}=\sigma_{a-b}\times V_{a}$$

where POTS is the test value of the instrument (MPa); σ_a−b_ is the adhesive strength between asphalt and aggregate (MPa); σ_a−s_ is the adhesive strength between asphalt and stub (MPa); A_a−b_ and A_a−s_ are the proportion of each failure form, respectively; σ_adh_ is the adhesive strength between FAM and aggregate (MPa); V_a_ is the volume fraction of asphalt in FAM.

There is also the cohesive failure inside the FAM. The pull-off tests of FAM specimens were conducted to obtain the cohesive strength of FAM. The gradation of FAM is consistent with Table 1. The two failure forms of the FAM pull-off tests are the same as the asphalt pull-off tests. One form was cohesive failure inside FAM when the FAM was both on the stub and basalt. The other form was adhesive failure between FAM and stub when there was no FAM on the stub and FAM on the basalt. The following formula was proposed to determine the cohesive strength of FAM:

(3) $$POTS=\sigma_{\mathrm{coh}}\times A_{m-b}+\sigma_{m-s}\times A_{m-s}$$

where POTS is the test value of the instrument (MPa); σ_coh_ is the cohesive strength inside FAM (MPa); σ_m−s_ is the adhesive strength between FAM and stub (MPa); A_m−b_ and A_m−s_ are the proportion of each failure form, respectively.

#### 3.2.2. SCB Tests

**Figure 3:**
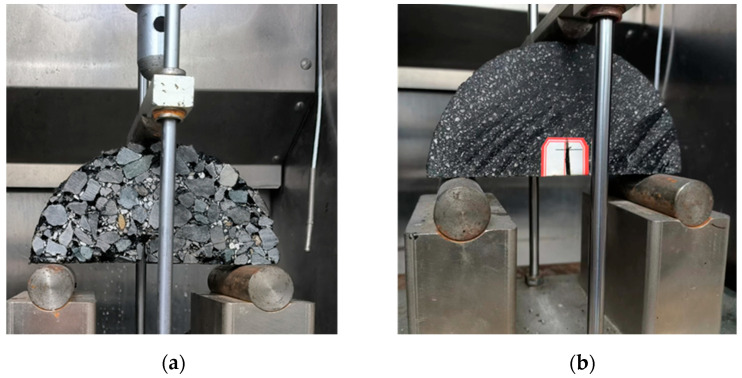
SCB tests setup. (**a**) PAC, (**b**) FAM.

SCB tests of PAC and FAM were conducted, respectively [37]. Superpave Gyratory Compactor (SGC) was utilized to make the PAC and FAM. The obtained cylindrical specimens were 150 mm in diameter and 150 mm in height. SCB specimens were cut from the central portion of the cylindrical samples to make the void content of specimens as close as possible to the target void content. These SCB specimens were 150 mm in diameter and 25 mm in thickness and had a notch with a depth of 15 mm and a width of 3 mm in the center. SCB tests were conducted in the laboratory using a universal testing machine (UTM-250) with an ambient chamber, as shown in Figure 3. Each specimen was placed in the ambient chamber for more than 4 h before the tests. The distance between the two supports is 120 mm, and the vertical loading rate was set at 5 mm/min.

The experimental results of the PAC were used to validate the model. The experimental results of the FAM provided the fracture energy *G_f_* as the parameters of FAM for subsequent study. The calculation formula for *G_f_* is defined as the fracture work W_f_ (the area enclosed by the load–displacement curve and the coordinate axes) divided by the ligament area (the product of the ligament length and thickness of the specimen) of the SCB specimen before testing [37], as follows:

(4) $$G_{f}=\frac{W_{f}}{A_{lig}}$$

(5) $$A_{lig}=\left(r-a\right)\times t$$

where G_f_ is fracture energy (J/m^2^); A_lig_ is ligament area (m^2^); r is specimen radius (m); a is notch length (m); t is specimen thickness (m).

#### 3.2.3. Preparation of Salt Condition Samples

Specimens of the pull-off tests and FAM specimens of the SCB tests were subjected to dry–wet (D–W) cycles and freeze–thaw (F–T) cycles using the salt solution to simulate a salt erosion environment on asphalt pavements in this study. The concentrations of salt solution used in the experiment were 0%, 5%, 10%, and 15%, respectively. Three parallel specimens were set up in each experimental group.

The process followed for the D–W cycles was as follows: soak the specimens in a solution at 25 °C for 12 h, then place them in a blast oven at 25 °C for 12 h, which was defined as a dry–wet cycle. Referring to [16,37], twelve dry–wet cycles were carried out in total.

The process followed for the freeze–thaw cycles was as follows:

(1) The specimens were conditioned in the salt solution by vacuum saturation using a residual pressure of 98 ± 0.3 kPa for 15 min.
(2) The specimens were placed in an ambient chamber with a temperature of −18 °C for 12 h.
(3) The frozen specimens were placed in a blast oven at 25 °C for 12 h. Since the FAM undergoes deformation at 60 °C, the dissolving temperature was set at 25 °C.

The above steps are defined as a freeze–thaw cycle. The freeze–thaw cycles were conducted 3, 6, and 9 times. It can be observed whether the increase in the number of F–T cycles can accelerate the effect of salt erosion. Specimens without any treatment were set as the control group. All specimens need to be placed in the ambient chamber with a temperature of −10 °C for 4 h before testing.

### 3.3. Experiment Results

The test results for the specimens after simulating the salt erosion environment are presented in Table 4. As shown in Table 4, under 0% salt solution concentration in the D–W cycles, the adhesive strength and cohesive strength decreased by 3.2% and 7.8%, respectively. When the concentration increased to 15%, the adhesive strength and cohesive strength decreased by 14.9% and 22.3%, respectively. This indicates that the salt solution could accelerate the deterioration of adhesive strength and cohesive strength. As the concentration increased from 0% to 15%, the *G_f_* of FAM decreased by 31.5%. The increase in salt solution concentration resulted in declines in the *G_f_* of FAM. On the one hand, this may be because the salt solution can corrode FAM and the chloride ions in the salt accelerate asphalt ageing, which also causes the deterioration of FAM performance. On the other hand, the crystallization of the salt solution within the FAM may cause expansion stress that damages the internal structure of the FAM during the drying phase. With increasing concentration, the expansion pressure intensifies, causing more damage to the FAM. 

As the concentration increased from 0% to 15%, the adhesive strength, cohesive strength, and *G_f_* of FAM decreased by 11.5%, 8.0%, and 15% at 3 F–T cycles, respectively. As the number of F–T cycles increased from 3 to 9, the adhesive strength, cohesive strength, and *G_f_* of FAM decreased by 11.7%, 8.9%, and 20.3% at a 15% concentration of salt solution, respectively. Both the increase in the number of F–T cycles and the increase in salt solution concentration lead to a noticeable decline in adhesive strength, cohesive strength, and *G_f_* of FAM, as shown in Table 4. The frost heave of water may lead to expansion pressure in the FAM, which could damage it. And the damage may accumulate with the increased number of F–T cycles. Like the D–W cycles, the salt solution and chloride ions also accelerate the deterioration of FAM.

## 4. Model Development and Validation

### 4.1. Development of SCB FE Model

Due to the high void content of PAC, the presence of voids is an inescapable factor when developing PAC models. This imparts complexity in developing the heterogeneous PAC models. In this study, an image-assisted approach was employed to develop a numerical model of PAC. Assuming that concrete is composed of three components: coarse aggregates, FAM, and voids, the specific process for model development is as follows:

(1) Data collection on the aggregate plane shape was conducted using the Aggregate Image System (AIMS). These projections were then subjected to image binarization.
(2) The binarized images were imported into discrete element software as templates. It was assumed that FAM uniformly covered the surface of the coarse aggregates. Following the specified gradation in Table 1, the FAM-encased aggregates were randomly placed without overlap in specific regions. Gravity was applied to all the aggregates to form the aggregate–aggregate contact network within the PAC, ensuring the transmission of forces. By performing the above options, the contour lines of the concrete section were obtained.
(3) The concrete of FAM and aggregates was proportionally reduced based on the gradation to obtain the contour lines of the aggregates.
(4) Finally, the contour lines of the concrete and aggregates were merged and converted into drawing exchange format (DXF) files. Then, these files were imported into finite element software to develop a heterogeneous PAC finite element model.

**Figure 4:**
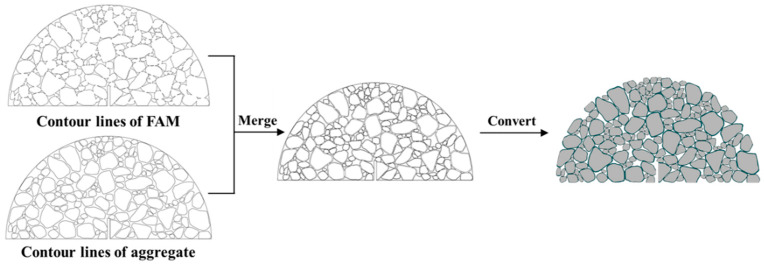
Development of heterogeneous model.

A schematic diagram of this process is illustrated in Figure 4.

### 4.2. Bilinear Cohesive Zone Model

The main causes of fracture in PAC are adhesive and cohesive failures. In this study, it is assumed that cracks occur at the FAM–aggregate interface and inside the FAM. This is because the stiffness and strength of the aggregate are higher than that of the asphalt and FAM. Moreover, the fracture of the aggregate typically occurs at very low temperatures, such as below −20 °C [38]. The SCB test temperature used in this study was −10 °C, which exceeds the temperature range proposed in the literature. So aggregate fracture is not considered in this study.

**Figure 5:**
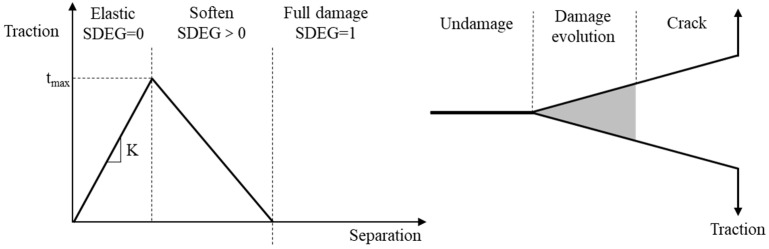
Bilinear cohesive model.

The bilinear cohesive zone model based on the traction–separation law is utilized to develop the numerical model, as shown in Figure 5. The effectiveness of the cohesive zone model (CZM) in studying the fracture behavior of concrete has been validated [39,40]. The model describes the linear elastic phase of the material before reaching its ultimate strength and the stiffness–softening phase after reaching its ultimate strength. Scalar stiffness degradation (SDEG) represents the degree of damage in the cohesive elements. The element is in the linear elastic phase before the traction force reaches its peak. At the same time, the SDEG value is 0. Once the traction force reaches its peak, the element moves from the linear elastic to the stiffness–softening phase, and the cohesive element begins to deteriorate, resulting in a gradual increase in SDEG. When the traction force decreases to zero at the maximum displacement and the SDEG reaches 1, the element fails, leading to cracking. As the number of failed cohesive elements increases, cracks start to develop within the concrete. The area enclosed by the line segment and the coordinate axis in Figure 5 represents the energy required for the failure of one element.

**Figure 6:**
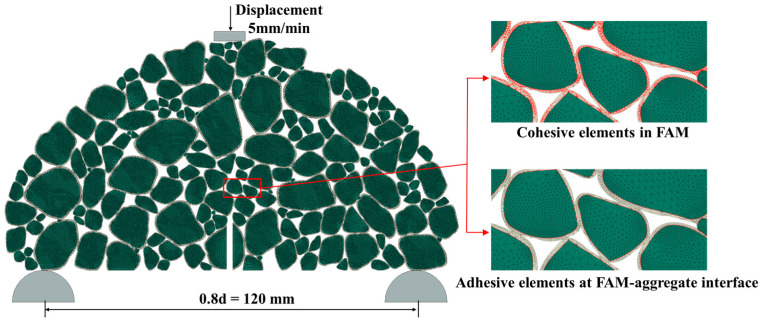
Example of the FE model.

In this study, zero-thickness cohesive elements were inserted at the asphalt–aggregate interface and within the FAM to represent damage evolution and crack propagation, as shown in Figure 6.

### 4.3. FE Model Parameters

In the model, the PAC is assumed to consist of coarse aggregate, FAM, and voids. Aggregates are defined as linear elastic materials, while FAM is defined as a viscoelastic material. The detailed parameters of FAM are represented by the Prony series, as shown in Table 5. The elastic modulus was tested by nano-indentation tests. The strength parameters of CZM elements can be obtained from pull-off tests conducted in the laboratory. The fracture energy of the cohesive elements in FAM can be obtained through SCB tests of FAM. A previous study has shown that the fracture energy of the FAM-aggregate interface is almost half of its of FAM [38]. It is challenging to determine the fracture energy of the FAM–aggregate interface element experimentally. Therefore, in this study, it is assumed to be half of the FAM fracture energy. The parameters of the CZM utilized in this study are outlined in Table 6.

In subsequent sections, the cohesive zone elements of FAM will be referred to as cohesive elements, and the cohesive zone elements of the aggregate–FAM interface will be referred to as adhesive elements, as shown in Figure 6.

The model utilized triangular elements for free meshing, maintaining a mesh density of 1 mm to balance computational accuracy and efficiency. The element types for aggregates and FAM were defined as CPS3 (three-node linear plane stress triangle elements). The average number of elements was approximately 200,000. The cohesive zone elements were defined as COH2D4 (four-node 2D cohesive elements), with an average of about 80,000 elements. The loading process of the SCB experiment was simulated by applying a vertical downward displacement load on the rigid indenter. The loading rate of the top indenter is 5 mm/min, which is the same as the SCB tests. In summary, the external conditions, model dimensions, and experimental loading conditions were replicated in the numerical model to closely match the laboratory SCB tests.

### 4.4. Model Validation

**Figure 7:**
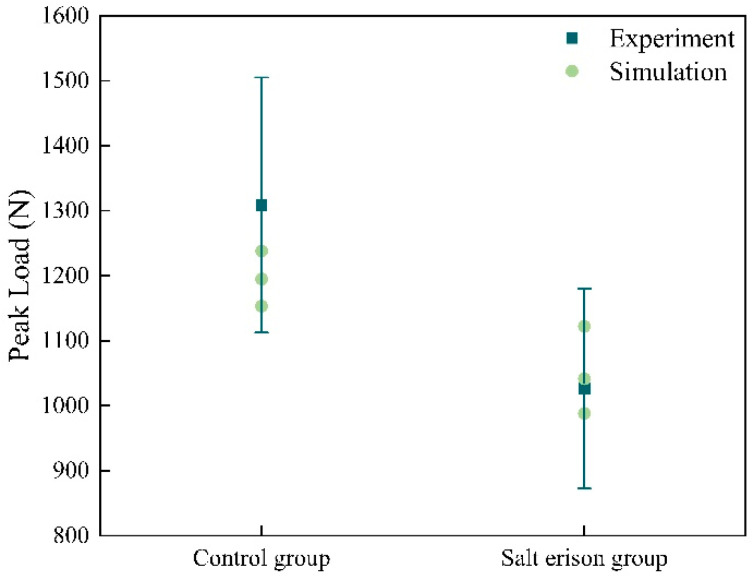
Comparison of SCB tests and numerical simulation.

Based on the laboratory SCB experiments and the CZM-based finite element models developed in this study, the peak load of PAC was obtained. Three models were developed in this study, and the validation against the experimental results is summarized in Figure 7. The salt erosion group selected three F–T cycles with a 15% concentration salt solution as an example. Using the 15% experiments as the error margin, it is evident that all simulation values fall within this margin of error. These relative differences indicate that the accuracy of the finite element models in this study is acceptable. Therefore, the CZM-based finite element model proposed in this study is reliable.

## 5. Simulation Results and Discussion

The finite element model proposed in this study has already been validated in previous sections. In this section, it is described how the results of the laboratory experiments are input into the validated finite element model for computation. This was done to analyze the effect on the fracture behavior of the PAC under a salt erosion environment. Furthermore, the influence of key factors including the loading rate, void content, adhesion and cohesion, strength, and energy are explored on the fracture behavior of the PAC.

### 5.1. Effect of Salt on Peak Load and G_f_

**Figure 8:**
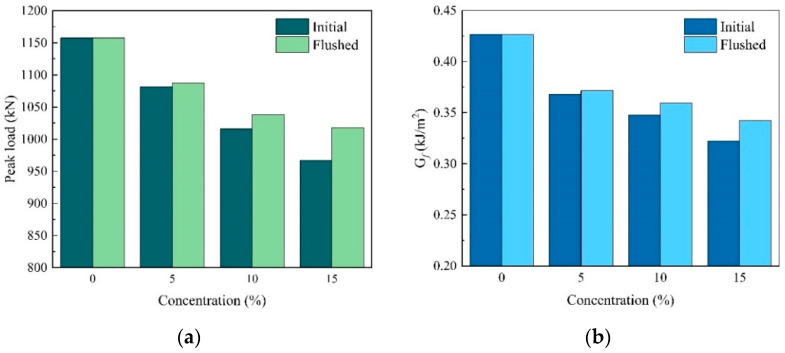
Simulation results of D–W cycles with different concentrations of salt solution: (**a**) peak load, (**b**) G*_f_*.

**Figure 9:**
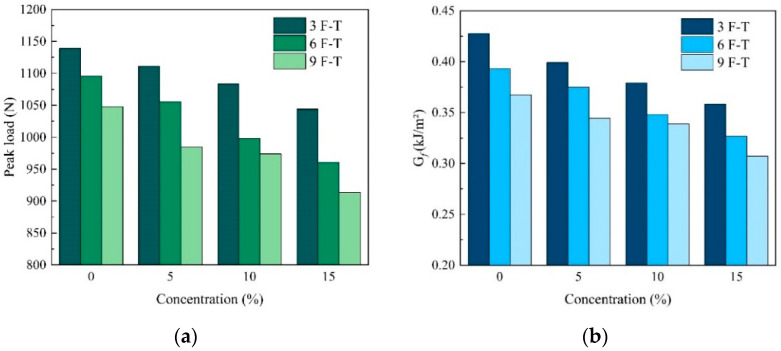
Simulation results of F–T cycles with different concentrations of salt solution: (**a**) peak load, (**b**) G*_f_*.

Based on the validated model and experiment results, the effect of salt erosion on the peak load and G*_f_* of the PAC were obtained. It is noteworthy that, to explore the methods of mitigating the influence of salt erosion on the PAC, a flushing treatment condition was used during the dry–wet cycle experiments. The flushing treatment consisted of immersing the specimen in salt solution for 12 h after each wet period and flushing the specimens with flowing water for approximately 20 s, simulating the flushing effect of a road sprinkler. The test results of the specimens after the flushing treatment were similarly used for the computational evaluations. The simulation results are shown in Figure 8 and Figure 9.

As shown in Figure 8, the peak load decreases by 5.5% and the fracture energy decreases by 8.2% on average for every 5% increase in salt solution concentration. It is evident that with an increase in the salt solution concentration, the peak load and fracture energy of PAC both show a decrease. This is because the strength and fracture energy of asphalt and FAM exhibit a decrease after salt erosion, which is proved by the experimental results as shown in Table 4. The main reason for the reduction in asphalt and FAM performance is that salt and chloride ions can accelerate the ageing of asphalt [13,16]. Moreover, salt also causes damage to the adhesive properties of the asphalt–aggregate interface [7,10], and the crystallization of salt during the drying phase generates expansion pressure which causes damage to the internal structure of the mixture [42]. The above damage resulted in a decrease in the PAC’s adhesive and cohesive properties, leading to a decline in its performance. Additionally, the influence of the flushing treatment is examined. At salt solution concentrations of 5%, 10%, and 15%, the peak load increases by 0.5%, 2.2%, and 5.3%, respectively, while the *G_f_* of the PAC increases by 0.9%, 3.4%, and 6.3%. This indicates that the flushing treatment before the drying phase can mitigate the decline in the PAC’s performance, and the effectiveness of the flushing treatment improves with higher salt solution concentrations. This could be attributed to the flushing effect, which lowers the concentration of the salt solution, consequently reducing the expansion pressure caused by salt crystallization during the drying phase. In seasonal frozen regions, large quantities of de-icing salt are used to prevent asphalt pavement from freezing during colder seasons. When there is no risk of freezing, removing the residual salt from the pavement through flushing treatment can alleviate the impact of salt erosion. Therefore, using equipment such as road sprinklers for flushing treatment after colder seasons can reduce the damage caused by the use of de-icing salt in seasonal frozen regions. 

As shown in Figure 9, with the concentration of salt solution increasing from 0% to 15%, the peak load and energy decreased by 8.36% and 16.19% after three freeze–thaw cycles. At the concentration of 15%, when the number of freeze–thaw cycles increased from three to nine, the load and energy decreased by 12.52% and 14.31%, respectively. This shows the same downward trend as the D–W cycles and both the salt solution and F–T cycles affect the performance of the PAC. The damage could have been caused by the expansion pressure generated during the freezing of water, which affects the internal structure of the specimen. Moreover, the salt solution and chloride ions also accelerate the deterioration of the FAM when the salt solution replaces pure water.

### 5.2. Effect of Salt on Scalar Stiffness Degradation (SDEG)

**Figure 10:**
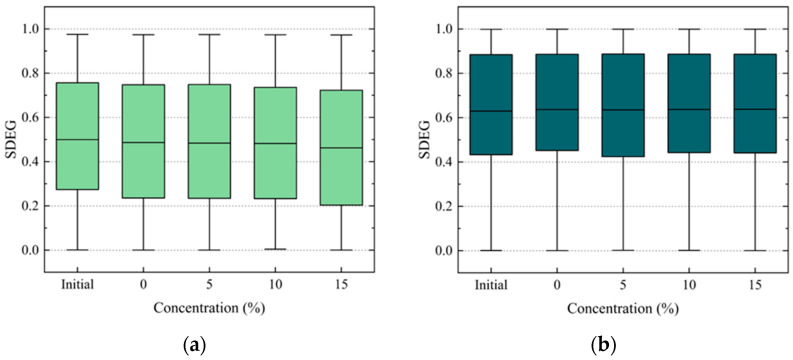
SDEG range of damaged elements at peak loads under D–W cycles: (**a**) adhesive elements, (**b**) cohesive elements.

**Figure 11:**
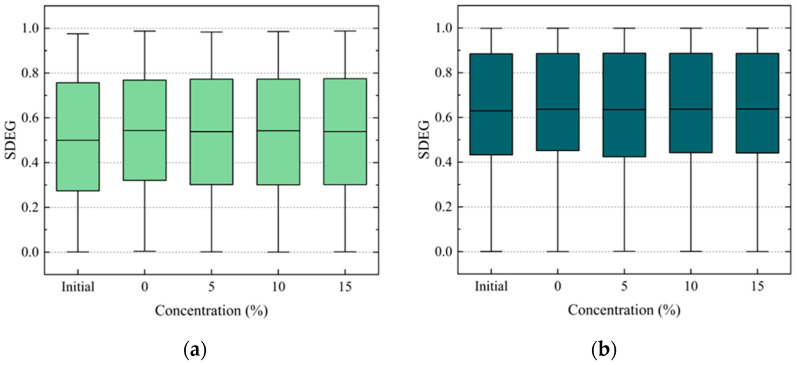
SDEG range of damaged elements at peak loads under 9 F–T cycles: (**a**) adhesive elements, (**b**) cohesive elements.

To study the damage to the internal structure of the PAC, the damage to the adhesive elements and cohesive elements was analyzed. The scalar stiffness degradation (SDEG) was analyzed to describe the distribution of the damage inside the PAC models. A larger SDEG value means more serious element damage. In numerical simulations, element damage is judged to be complete when the SDEG reaches a critical value. In this paper, this value was taken as 1. The distribution of the SDEG at peak load under different conditions is shown in Figure 10 and Figure 11. 

As shown in Figure 10a, the average value of the SDEG of the adhesive elements with 15% salt solution decreased by 4.9% compared to that with pure water. At the same time, the average value of the SDEG of the cohesive elements with 15% salt solution decreased by 0.3% compared to that with pure water, as shown in Figure 10b. There is a small tendency for the average SDEG valuesof adhesive elements to decrease with increasing salt solution concentration (Figure 10a) while the average SDEG values of the cohesive elements remain almost constant (Figure 10b). This could be attributed to the initial conditions, where both the adhesive and cohesive elements share the external load. As the salt solution concentration increases, the cohesive strength decreases faster than the adhesive strength. The reduced strength of the cohesive elements leads to them being able to withstand less stress, causing earlier failure of these elements. Due to this earlier failure, the cracks spread rapidly. Consequently, the stress transferred to the adhesive elements from the cohesive elements decreases, resulting in reduced damage to the adhesive elements.

The average SDEG value of the adhesive elements with 15% salt solution increased by 7.2% compared to that of the control group, as shown in Figure 11a. The average SDEG value of the cohesive elements with 15% salt solution increased by 1.2% compared to the control group, as shown in Figure 11b. The damage of the adhesive-damaged elements subjected to the F–T cycles is more serious than it is under the initial condition, which indicates that the coupling effect of the salt and F–T cycles leads to more serious damage to the adhesive elements. The SDEG of the cohesive elements subjected to the F–T cycles is almost unchanged. However, it is different from that of the adhesive elements subjected to the D–W cycles. This may be because the adhesive strength decreases faster with the F–T cycles than in the D–W cycles. As shown in Table 4, with the increase in the concentration of the salt solution, the gap between the adhesive strength and cohesive strength gradually decreases. This gap remains largely unchanged during the F–T cycles. Therefore, there was a small decreasing trend in the damaged adhesive elements subjected to theincreasing concentration of the salt solution along with the D–W cycles, while there was increasing trend in the samples subjected to the F–T cycles. 

**Figure 12:**
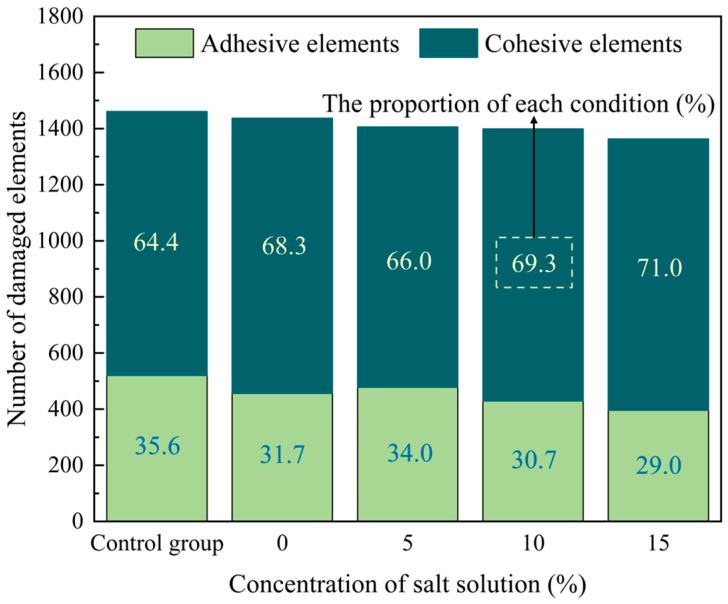
The number and proportion of damaged elements under D–W cycles.

Moreover, comparing Figure 10 and Figure 11, it is evident that under each concentration of salt solution, the damage to the adhesive elements is consistently less than that of the cohesive elements. The number and proportion of damaged elements subjected to different concentrations of salt solution and D–W cycles were statistically analyzed, as shown in Figure 12. It can be observed that the proportion of damaged cohesive elements of all the damaged elements is greater than that of the damaged adhesive elements under each condition. Moreover, this proportion of damaged cohesive elements generally exhibits an upward trend with the increase in the salt solution concentration. This may be attributed to the fact that the FAM serves as the primary component bearing external loads in the PAC. This conjecture will be further investigated in subsequent sections.

### 5.3. Effect of Void Content on Damage Type of Elements

**Figure 13:**
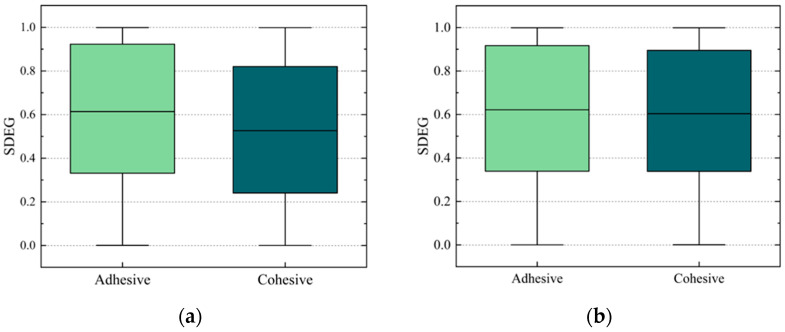

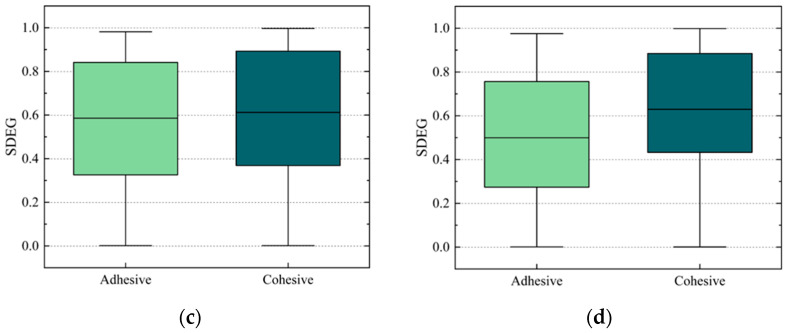
SDEG distribution with different void contents: (**a**) 0%, (**b**) 6%, (**c**) 12%, (**d**) 18%.

From the previous sections, it is evident that the damage caused to the adhesive elements is smaller than the damage caused to the cohesive elements under different concentrations of salt solution. This indicates that the damaged elements are primarily the cohesive elements in the fracture of the PAC. On the contrary, the damage to the adhesive elements is greater than that to the cohesive elements and the interface is the weaker part at the peak load in the dense-graded concrete [41]. The results for both are contrasting. Therefore, the effect of the void content on the damaged adhesive elements and cohesive elements is studied in this section. The simulated results are shown in Figure 13.

**Figure 14:**
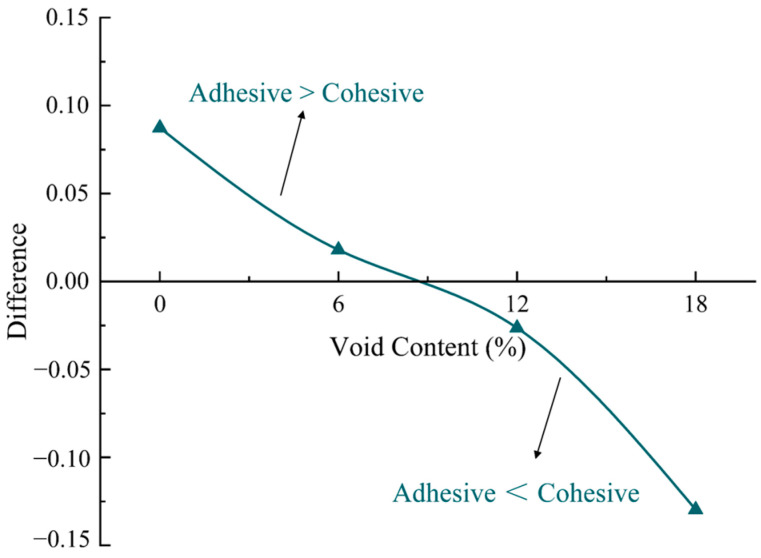
Difference in the average SDEG values between adhesive and cohesive elements.

**Figure 15:**
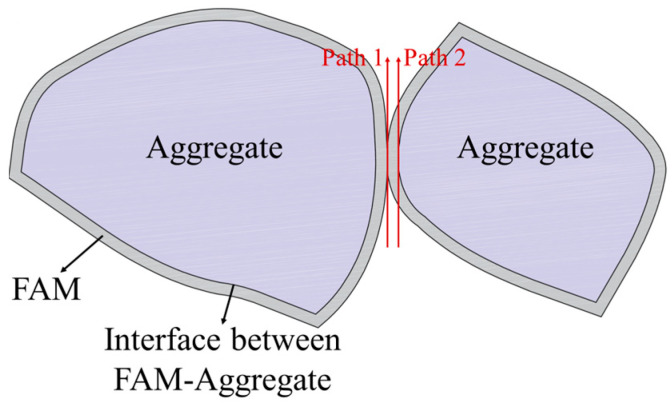
Schematic of two possible crack propagation paths.

As shown in Figure 13, the average SDEG values of the cohesive elements decreased with increasing void content, while the average SDEG values of the cohesive elements increased. Furthermore, the difference in the average SDEG values between the adhesive and cohesive elements was computed, and the results are illustrated in Figure 14. As the void content increases, the average SDEG value of the cohesive elements increases and surpasses the average SDEG value of the adhesive elements at a void content of approximately 9%. The interface strength is slightly less than the FAM strength [38,39]. When the void content is low, the cracking path tends to propagate along the interface due to the lower interface strength. After sufficient mixing, each aggregate will be covered by FAM, as shown in Figure 15. As the void content increases, the number of voids within the model gradually increases. Previous studies have indicated that the cracking path tends to propagate along the direction with more voids [43]. Compared with Path 2, Path 1 of the crack is more consistent with the propagation along the void–void direction. Therefore, Path 1 tends to crack first during the process of loading. This becomes more common with the increase in the void content. Consequently, as the void content increases, the cohesive damage becomes more severe. The cohesive elements on Path 1 have completely failed, and the adhesive and cohesive elements on Path 2 have also been damaged, but the average SDEG value of the damaged adhesive elements is smaller than that of the cohesive elements. This suggests that more emphasis should be placed on the performance of FAM to prevent the occurrence of cracking in the design of PAC. 

### 5.4. Effect of Cohesion and Adhesion on Fracture Behavior

**Figure 16:**
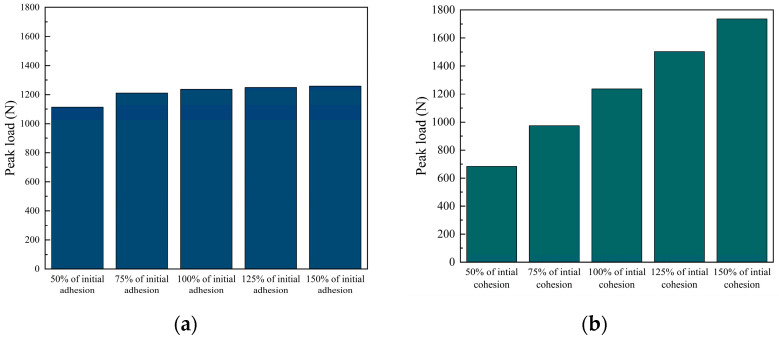
Effect of adhesion and cohesion on peak load: (**a**) adhesion change, (**b**) cohesion change.

Previous sections have highlighted the influence of the cohesive property on PAC. In this section, the effects of the properties of the adhesive and cohesive elements will be analyzed on the fracture behavior. Two simulation approaches were employed. Firstly, the performance (including strength and fracture energy) of the adhesive elements (hereinafter referred to as adhesion) was kept constant and the performance of the cohesive elements (hereinafter referred to as cohesion) varied to 50%, 75%, 125%, and 150% of their initial values. Secondly, the cohesion was kept constant and the adhesion was varied to 50%, 75%, 125%, and 150% of their initial values. All other conditions of the model remained unchanged. The simulation results are depicted in Figure 16.

As shown in Figure 16a, for every 10% increase in adhesion, the specimen can bear 1.18% more load. It can be observed that with every 10% increase in cohesion, the specimen can bear 8.51% more load, as shown in Figure 16b. In summary, enhancing both cohesion and adhesion can increase the peak load of the PAC. However, cohesion has a greater impact on the peak load compared to adhesion. This conclusion confirms the earlier conjecture. It indicates that the performance of FAM has a much greater impact on the durability of PAC than the FAM–aggregate interface. This suggests that more emphasis should be placed on the performance of FAM in the design of PAC.

**Figure 17:**
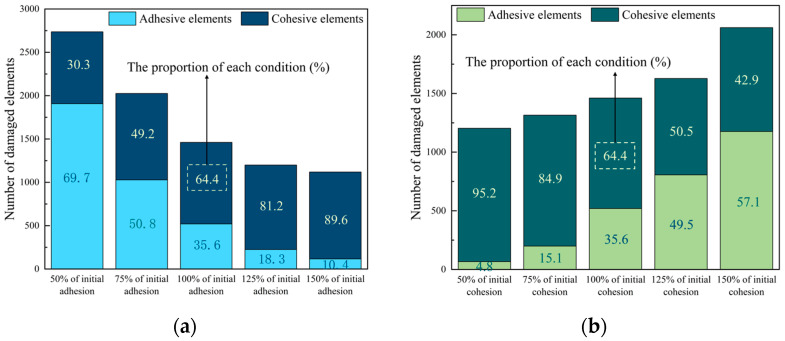
Effect of adhesion and cohesion on the number and proportion of damaged elements: (**a**) adhesion change, (**b**) cohesion change.

Furthermore, Figure 17 shows the effect of adhesion and cohesion on the number and proportion of damaged elements. 

When the adhesion increases, the number and proportion of damaged adhesive elements decrease, as shown in Figure 17a. This is because adhesion increases but the peak load changes little, as shown in Figure 16a, and fewer adhesive elements can bear the external load. The increase in adhesion also leads to more damaged cohesive elements, as shown in Figure 17a. The reason for this is that the increase in adhesion causes a relatively lower strength of FAM compared to the interface. When the FAM is relatively weaker than the interface, the crack path is more likely to propagate around the FAM. On the contrary, the number and the proportion of damaged adhesive elements increase with the increase in cohesion, as shown in Figure 17b. This is because the crack path is more likely to propagate around the interface, which is the weaker part. When the cohesion increased from 50% to 150% of the initial cohesion, the number of damaged cohesive elements decreased by only 33.8%. At the same time, there are more adhesive elements to bear the load, so the peak load increases with the increase in cohesion. 

### 5.5. Effect of Salt on Loading Rate

**Figure 18:**
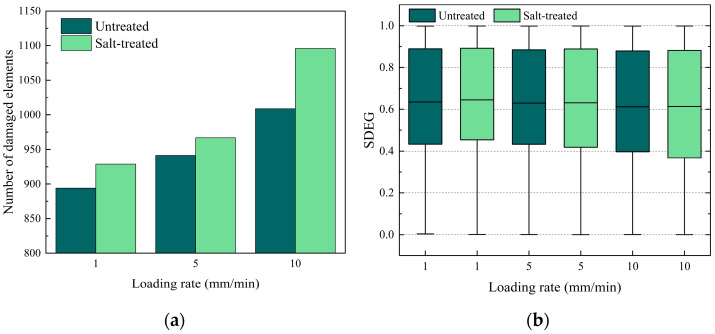
Effect of salt erosion and loading rate on fracture behavior: (**a**) number of cohesive damaged elements, (**b**) SDEG distribution of cohesive elements.

To investigate the influence of salt erosion on the loading rate, three different loading rates, 1 mm/min, 5 mm/min, and 10 mm/min, were considered. The strength and G*_f_* of the control group and 15% concentration salt solution and D–W cycle group were used for evaluating the influence of salt erosion on the loading rate. Other parameters of the numerical simulation were kept the same as those used in the model validation. The simulation results are shown in Figure 18. 

As shown in Figure 18a, it is observed that the number of damaged elements increased under both the untreated and salt−treated groups with the increase in the loading rate. When the loading rate increased from 1 mm/min to 10 mm/min, the average SDEG value decreased by 3.6%, as shown in Figure 18b. It indicates that the average SDEG value decreases with the increase in the loading rate. This is because the stiffness of the cohesive elements is higher at higher loading rates, and it cannot bear more stress through deformation. This leads to the excessive stress being transferred to more elements and borne by these elements together. In general, the increase in the loading rate may result in more but minor element damage. Furthermore, the number of damaged cohesive elements in the salt-treated group is more than that in the untreated group, as shown in Figure 18a. After the salt erosion, the average SDEG values increased by 1.6%, 0.2%, and 0.4% at 1, 5, and 10 mm/min loading rates, respectively. This indicates that salt erosion leads to more and severer cohesive element damage under each loading rate, which also indicates the deterioration of PAC durability due to salt erosion.

## 6. Conclusions

In this study, a CZM-based FE model was developed to evaluate the effect of moisture and salt erosion on the fracture behavior of PAC. Numerical simulations of SCB tests were conducted, and the accuracy of the model was validated by the results of laboratory SCB tests. With the validated model, the effects of salt erosion and some crucial factors on the fracture behavior of PAC were investigated. The following conclusions were reached from the analysis:

(1) Salt solution accelerates the degradation of asphalt and FAM when subjected to both dry–wet cycle and freeze–thaw cycle conditions. Under 15% salt solution with dry–wet cycles, the adhesive strength and cohesive strength decreased by 14.9% and 22.3% compared to the control group. As the concentration increased from 0% to 15%, the G*_f_* of the FAM decreased by 31.5%. The salt erosion, chloride ions, and expansion pressure caused by salt crystallization can accelerate the deterioration of asphalt and FAM in salt environments.
(2) Dry–wet cycles and freeze–thaw cycles contribute to the deterioration of the PAC. Salt erosion can accelerate this degradation. Flushing treatment before the drying phase can mitigate the impact of salt erosion. This method helps reduce the effects of de-icing salt used for PAC pavements in seasonal frozen areas.
(3) The average SDEG values of the cohesive elements were higher than that of the adhesive elements during the fracture of the PAC. The damaged elements primarily were cohesive elements. This stands in contrast to situations observed in dense-graded asphalt mixture. As the void content increased, the average SDEG value of the cohesive elements increased and surpassed the average SDEG value of the adhesive elements at a void content of approximately 9%.
(4) An increase in cohesion is better for enhancing the fracture resistance of PAC, compared to adhesion. The relative magnitudes of the adhesion and cohesion forces can influence the crack propagation path.
(5) With an increase in the loading rate, the number of damaged elements increased, and the average value of the SDEG decreased under both the untreated group and the salt-treated group. Salt erosion resulted in a higher average SDEG value and a larger number of damaged cohesive elements at each loading rate.

## Figures and Tables

**Table 1:** Gradation of the PAC full mix and FAM [35].

Sieve Size (mm)		16	13.2	9.5	4.75	2.36	1.18	0.6	0.3	0.15	0.075
Passing percentage (%)	PAC	100	85.5	58.2	21.0	8.6	5.7	4.7	3.7	2.1	2.1
	FAM	100	100	100	100	100	100	82.5	64.9	36.8	36.8

**Table 2:** Physical properties of 70# asphalt binder.

Penetration (25 °C, 0.1 mm)	Ductility (15 °C, cm)	Softening Point (°C)	Density (g/cm^3^)
70.0	>100	47.5	1.0

**Table 3:** Physical properties of basalt.

Apparent Density (g/cm^3^)	Water Absorption (%)	Crushing Value (%)	Abrasion Value (%)
2.7	0.9	16.0	20.1

**Table 4:** Experiment results of pull-off tests and SCB tests of FAM.

Tests Conditions	Cycles	Concentration (%)	Adhesive Strength (MPa)	Cohesive Strength (MPa)	*G_f_* of FAM (kJ/m^2^)
Control group	0	0	6.80	7.71	3.57
Dry–wet	12	0	6.58	7.11	3.36
		5	6.12	6.79	2.71
		10	5.90	6.28	2.56
		15	5.79	5.99	2.30
Freeze–thaw	3	0	6.08	7.09	3.13
		5	5.60	6.92	3.02
		10	5.46	6.77	2.84
		15	5.38	6.52	2.66
	6	0	5.82	6.79	2.99
		5	5.43	6.55	2.80
		10	5.39	6.18	2.54
		15	5.12	5.99	2.30
	9	0	5.44	6.53	2.71
		5	5.15	6.09	2.52
		10	5.07	6.03	2.46
		15	4.75	5.94	2.12

**Table 5:** Parameters for the Prony series [41].

Temperature (°C)	*g*_1_	*τ*_1_	*g*_2_	*τ*_2_
−10	0.09	5.11	0.84	74.37

**Table 6:** Parameters for CZM.

Phase	E (MPa)	Poisson’s Ratio [41]	σ (MPa)	G_I_ (kJ/m^2^)
Aggregate	55,500	0.15		
FAM	1200	0.25	7.71	3.57
Interface	1200	0.25	6.80	1.79

## Data Availability

Data are contained within the article.

## References

[1] Cappellesso V.G., Van Mullem T., Gruyaert E., Van Tittelboom K., De Belie N. (2023). Bacteria-based self-healing concrete exposed to frost salt scaling. Cem. Concr. Compos..

[2] Das J.K., Pradhan B. (2023). Significance of chloride salt type and sulfate salt on chloride transport mechanism of concrete in the presence of corrosion inhibiting admixtures. Constr. Build. Mater..

[3] Li H., Zhang Y., Guo H. (2021). Numerical simulation of the effect of freeze–thaw cycles on the durability of concrete in a salt frost environment. Coatings..

[4] Golewski G.L. (2023). The Effect of the addition of Coal Fly Ash (CFA) on the control of water movement within the structure of the concrete. Materials.

[5] Mączka E., Mackiewicz P. (2023). Asphalt mixtures degradation induced by water, frost, and road salt in the 4-PB bending test evaluated by stiffness variability. Road Mater. Pavement.

[6] Ogbon W.A., Jiang W., Yuan D., Xing C., Xiao J. (2024). Asphalt mixture performance deterioration in the salty environment based on theoretical calculation. Constr. Build. Mater..

[7] Jiang Q., Liu W., Wu S., Gou X. (2024). Study on the mechanical performance damage in laboratory-simulated periodic salt environment for asphalt concrete. Constr. Build. Mater..

[8] Fakhri M., Javadi S., Sedghi R., Arzjani D., Zarrinpour Y. (2019). Effects of deicing agents on moisture susceptibility of the WMA containing recycled crumb rubber. Constr. Build. Mater..

[9] Mączka E., Mackiewicz P. (2022). Asphalt mixtures and flexible pavement construction degradation considering different environmental factors. Appl. Sci..

[10] Juli-Gándara L., Vega-Zamanillo Á., Calzada-Pérez M.Á. (2019). Sodium chloride effect in the mechanical properties of the bituminous concrete. Cold Reg. Sci. Technol..

[11] Jeon I., Lee J., Lee T., Yun T., Yang S. (2024). In silico simulation study on moisture-and salt water-induced degradation of asphalt concrete mixture. Constr. Build. Mater..

[12] Zhang Q., Huang Z. (2019). Investigation of the microcharacteristics of asphalt mastics under dry-wet and freeze-thaw cycles in a coastal salt environment. Materials.

[13] Guo R., Zhang H., Tan Y. (2022). Influence of salt dissolution on durable performance of asphalt and self-ice-melting asphalt concrete. Constr. Build. Mater..

[14] Zhang R., Tang N., Zhu H. (2022). The effect of sea salt solution erosion on cohesion, chemical and rheological properties of SBS modified asphalt. Constr. Build. Mater..

[15] Ogbon W.A., Xu H., Jiang W., Xing C. (2023). Polymer-modified asphalt binders’ properties deterioration under the action of chloride salt. Road Mater. Pavement Des..

[16] Zhang K., Luo Y., Li Z., Zhao Y., Zhao Y. (2022). Evaluation of performance deterioration characteristics of asphalt concrete in corrosion environment formed by snow-melting agents. J. Mater. Civ. Eng..

[17] Chen X., Ren D., Tian G., Xu J., Ali R., Ai C. (2023). Influence of salt dissolution on durable performance of asphalt and self-ice-melting salt and acid erosion environments based on multi-scale analysis. Constr. Build. Mater..

[18] Shadman M., Ziari H. (2017). Laboratory evaluation of fatigue life characteristics of polymer modified porous asphalt: A dissipated energy approach. Constr. Build. Mater..

[19] Manrique-Sanchez L., Caro S. (2019). Numerical assessment of the structural contribution of porous friction courses (PAC). Constr. Build. Mater..

[20] Shahnewaz S.M., Masri K.A., Ghani N.A.A.A., Jaya R.P., Choo C.S., Giannakopoulou P.P., Petrounias P. (2023). Porous asphalt mixtures enriched with bamboo fibers as a new approach for future sustainable construction. Constr. Build. Mater..

[21] Wu H., Yu J., Song W., Zou J., Song Q., Zhou L. (2020). A critical state-of-the-art review of durability and functionality of open-graded friction course mixtures. Constr. Build. Mater..

[22] Limón-Covarrubias P., Avalos Cueva D., Valdés Vidal G., Reyes Ortiz O.J., Adame Hernández R.O., Galaviz González J.R. (2019). Analysis of the behavior of sma mixtures with different fillers through the semicircular bend (SCB) fracture tests. Materials.

[23] Yee T.S., Hamzah M.O. (2019). Evaluation of moisture susceptibility of asphalt-aggregate constituents subjected to direct tensile test using imaging technique. Constr. Build. Mater..

[24] Mihandoust M., Ghabchi R. (2023). Mechanical, thermodynamical, and microstructural characterization of adhesion evolution in asphalt binder-aggregate interface subjected to calcium chloride deicer. Constr. Build. Mater..

[25] Chaturabong P., Bahia H.U. (2018). Effect of moisture on the cohesion of asphalt mastics and bonding with surface of aggregates. Road Mater. Pavement.

[26] Daryaee D., Vamegh M. (2022). Feasibility Study for Evaluating the Moisture Resistance of Asphalt Mixtures Containing RAP Using Pull-off Test. J. Test. Eval..

[27] Motevalizadeh S.M., Sedghi R., Rooholamini H. (2020). Fracture properties of asphalt concrete containing electric arc furnace slag at low and intermediate temperatures. Constr. Build. Mater..

[28] Guo Q., Li G., Gao Y., Wang K., Dong Z., Liu F., Zhu H. (2019). Experimental investigation on bonding property of asphalt-aggregate interface under the actions of salt immersion and freeze-thaw cycles. Constr. Build. Mater..

[29] Wang X., Ren J., Gu X., Li N., Tian Z., Chen H. (2021). Investigation of the adhesive and cohesive properties of asphalt, mastic, and FAM in porous asphalt concrete. Constr. Build. Mater..

[30] Zokaei M., Hesami S. (2024). Prediction of fatigue crack behaviour of carbon fibre reinforced asphalt using fracture testing and modelling of the adhesive zone 50. Int. J. Pavement Eng..

[31] Khan Z.H., Amanul Hasan M., Tarefder R.A. (2022). Phase field approach to damage and fracture in asphalt concrete using multiscale finite element modeling of an instrumented pavement section. Eng. Fract. Mech..

[32] Caro S., Manrique-Sanchez L., Kim Y. (2021). Computational evaluation of long-term ravelling susceptibility of permeable friction courses (PAC). Constr. Build. Mater..

[33] Manrique-Sanchez L., Caro S., Arámbula-Mercado E. (2018). Numerical modelling of ravelling in porous friction courses (PAC). Road Mater. Pavement Des..

[34] Manrique-Sanchez L., Caro S., Arámbula-Mercado E. (2022). Random generation of 2D PAC microstructures through DEM gravimetric methods. Road Mater. Pavement Des..

[35] Manrique-Sanchez L., Caro S., Estrada N., Castillo D., Alvarez A.E. (2022). Coupled effects of ageing and moisture on the fracture properties of permeable friction courses (PFC). Int. J. Pavement Eng..

[36] (2017). Standard Tests Method for Pull-Off Strength of Coatings Using Portable Adhesion Testsers.

[37] (2015). Standard Method of Tests for Determining the Fracture Energy of Asphalt Mixtures Using the Semi-Circular Bend Geometry (SCB).

[38] Yin A., Yang X., Zeng G., Gao H. (2015). Experimental and numerical investigation of fracture behavior of asphalt concrete under direct shear loading. Constr. Build. Mater..

[39] Teng G., Zheng C., Chen X., Lan X., Zhu Y., Shan C. (2021). Numerical fracture investigation of single-edge notched asphalt concrete beam based on random heterogeneous FEM model. Constr. Build. Mater..

[40] Trawiński W., Bobiński J., Tejchman J. (2016). Two-dimensional simulations of concrete fracture at aggregate level with cohesive elements based on X-ray μCT images. Eng. Fract. Mech..

[41] Chen J., Ouyang X., Sun X. (2022). Numerical investigation of asphalt concrete fracture based on heterogeneous structure and cohesive zone model. Appl. Sci..

[42] Zhang K., Luo Y., Xie W., Wu J. (2020). Evaluation of road performance and adhesive characteristic of asphalt binder in salt erosion environment. Mater. Today Commun..

[43] Zhang H., Ding H., Rahman A. (2022). Effect of asphalt mortar viscoelasticity on microstructural fracture behavior of asphalt mixture based on cohesive zone model. J. Mater. Civ. Eng..

